# Mental health attendances in Australia during the COVID-19 pandemic: A telehealth success story?

**DOI:** 10.1192/j.eurpsy.2021.1848

**Published:** 2021-08-13

**Authors:** S. Giles, S. Sreedharan, M. Mian

**Affiliations:** 1 Melbourne School Of Psychological Sciences, The University of Melbourne, Parkville, Australia; 2 Melbourne Medical School, The University of Melbourne, Parkville, Australia

**Keywords:** COVID-19, telehealth, mental health

## Abstract

**Introduction:**

The COVID-19 pandemic has significantly impacted the delivery of mental health services globally. Within Australia, the COVID-19 pandemic and subsequent containment measures have led to reduced face-to-face attendances. To maintain access to mental health consultations, new telehealth services were introduced by the Australian Government in late March 2020.

**Objectives:**

We aimed to quantify the impact of the COVID-19 pandemic on patterns of mental health attendances in Australia using an interrupted time series model.

**Methods:**

To characterise patterns of mental health service utilisation, monthly mental health attendances between January 2016 and June 2020 were extracted from the Medicare database, stratified by clinician type: general practitioner (GP), psychiatrist, and allied health. We used triple exponential smoothing to model attendances between January 2017 and December 2019. Observed and predicted attendances between January and June 2020 were compared with 95% confidence (p<0.05).

**Results:**

Our models showed decreased mental health attendances in March and April, consistent with all healthcare services during this time. While uptake of telehealth was significant, it only partially covered the reduction in mental health attendances.

**Conclusions:**

Our modelling highlights the significant impacts of the COVID-19 pandemic on mental health services in Australia, with telehealth only partially compensating for the reduction in face-to-face attendances. These results suggest that telehealth services may not be suitable for all individuals (e.g. those without reliable internet access). Given that telehealth will likely remain a feature of mental health service provision, outreach and face-to-face services should be considered for vulnerable groups

**Disclosure:**

No significant relationships.

